# Tubal factor, cleavage stage and more than one embryo transferred were risk factors associated with ectopic pregnancy after assisted reproductive treatment

**DOI:** 10.5935/1518-0557.20210074

**Published:** 2022

**Authors:** Vanessa Devens Trindade, Marta Ribeiro Hentschke, Victória Campos Dornelles, Juliana Ferri-Guerra, Ariane Tieko Frare Kira, Talita Colombo, Alvaro Petracco, Rafaella Gehm Petracco, Joao Michelon, Bartira Ercilia Pinheiro Da Costa, Mariangela Badalotti

**Affiliations:** 1 Pontifical Catholic University of Rio Grande do Sul, PUCRS. Graduate Program of Medicine and Health Sciences. Porto Alegre, Rio Grande do Sul, Brazil; 2 Fertilitat - Center for Reproductive Medicine. Medical Department. Porto Alegre, Rio Grande do Sul, Brazil; 3 Mount Sinai Medical Center, Department of Internal Medicine, Miami, USA

**Keywords:** Ectopic pregnancy, *in vitro* fertilization, risk factors

## Abstract

**Objective:**

Ectopic pregnancy is a well-known complication following in vitro fertilization with embryo transfer; studies have questioned, however, whether there are risk factors that could be identified before the procedure. The objective of this study was to investigate the possible risk factors involved in ectopic pregnancy following *in vitro* fertilization.

**Methods:**

Retrospective case-control study performed at an assisted reproduction clinic in Brazil. To select the control group, we used a 1:4 ratio. The study included 499 patients submitted to in vitro fertilization with clinical pregnancy. We collected the data from electronic records, between 2000-2019 and divided into: Group 1, ectopic pregnancy (n=90) and Group 2, intrauterine pregnancies (n=409).

**Results:**

When comparing groups, the results observed were: Tubal factor infertility (35.6% vs. 21.1%, *p*=.005) (OR 2.0 [1.2-3.4], *p*=.004); Previous miscarriage history (15.1% *vs*. 7.1%, *p*<.05) (OR 2.0 [1.02-4.29], *p*=.044); Number of cleavage-stage embryo transfers (69.2% *vs*. 54.0 *p*=.028) (OR 1.9 [1.08-3.33], *p*=.025); Two or more embryos transferred (OR 2.5 [1.12-5.70], *p*=.025), all associated with greater ectopic pregnancy risk. Oocyte recipient patients were excluded from this analysis, but when included a difference was found when comparing the groups (9.4% (10/106) *vs*. 3.0% (13/434), *p*=.007), (OR 3.3 [1.41-7.98] *p*=.005); this result should be interpreted with caution because of the sample size.

**Conclusions:**

In high-risk patients, a single blastocyst transfer seems to be a reasonable approach to decrease the ectopic pregnancy risk.

## INTRODUCTION

Ectopic pregnancy (EP) is an obstetrical disorder potentially associated with maternal death in the first trimester (Refaat et al., 2015; Zhang et al., 2017). It is one of the well-known complications following in vitro fertilization (IVF) with embryo transfer (ET) (Cheng et al., 2015; Bu et al., 2016; Liu et al., 2019). Studies showed that 95% of EP occurs in the fallopian tubes, but there are reports of interstitial pregnancy, cornual pregnancy, cervical pregnancy and ovarian pregnancy (Liu et al., 2019). The incidence of EP in spontaneous pregnancies is close to 1-2%, while in IVF cycles it reaches 0.9-11% (Refaat et al., 2015; Muller et al., 2016; Liu et al., 2019). This variation could be explained by different denominators. Pelvic inflammatory disease, tubal pathology, previous pelvic surgery, and uterine cavity abnormalities have been defined as factors that mostly contribute to the risk of EP in infertile women (Cheng et al., 2015). The etiology of a higher EP rate in IVF-ET cycles, compared to natural conception, remains unclear (Kim et al., 2019). Several hypotheses have been suggested to explain this difference, including stimulation protocol, ET strategies, reproductive health characteristics of infertile women, estimated embryo implantation potential and laboratory techniques (Cheng et al., 2015; Liu et al., 2019). Even though strategies to decrease EP rates are limited, it is important to accurately identify its risk factors.

Ectopic pregnancy after IVF represents not only a life-threatening event, but also a lost opportunity for couples who sought assisted reproductive technique (ART) treatment (Londra et al., 2016). Thus, the objective of the present study was to investigate the possible risk factors for EP following IVF.

## MATERIALS AND METHODS

A retrospective case-control study was performed at Fertilitat - Reproductive Medicine Center, between January 2000 and August 2019.

### Study Population

**Figure 1:**
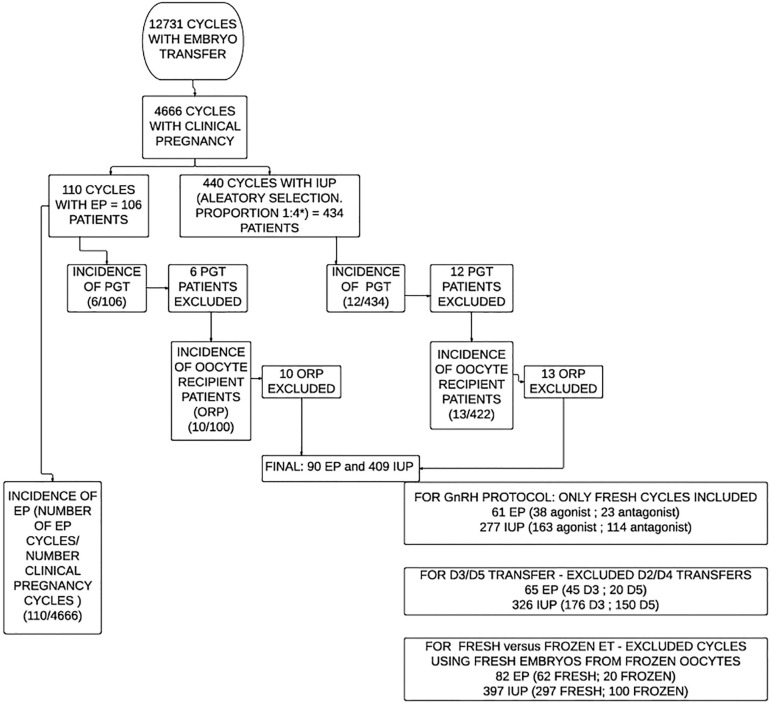
Study population design

Patients that underwent ET were initially selected for the study, which included 12.731 transfer cycles during the proposed period of study. Of those, 4.666 cycles resulted in a clinical pregnancy following at least one ET. EP occurred in 106 patients (110 ET cycles). The control group was randomly selected using a statistical analysis program with a ratio of 1:4. Only the first EP or intrauterine pregnancy (IUP) cycles were included. Preimplantation genetic test (PGT) cycles and oocyte recipient patients (ORP) were excluded from the main analysis. Figure 1 summarizes the study population and design.

EP: Ectopic pregnancy, IUP: intra uterine pregnancy, PGT: preimplantation genetic test, ORP: oocyte recipient patients, ET: embryo transfer. *use of SPSS Statistical Analysis Software.

Finally, the sample was divided into two groups: Group 1, EP (n=90) and Group 2, IUP (n=409). To analyze PGT rates between groups, another analysis was carried out, which included 106 patients in Group 1 and 434 patients in Group 2. Likewise, to analyze ORP rates between groups, another analysis was performed including 100 patients in Group 1 and 422 in Group 2.

### Sample size

The power was calculated to be 80% with a 95% confidence interval, assuming a mean (%) difference of 14% in tubal infertility history between the EP and IUP groups, respectively. The control group was randomly selected using a statistical analysis program with a ratio of 1:4.

### Definitions for study groups

The IUP was defined as a pregnancy with one or more intrauterine gestational sacs confirmed by an ultrasound image. The EP was defined as the presence of an extrauterine gestational sac visualized on ultrasound or following surgical intervention, or also the absence of an intrauterine gestational sac with abnormal increased serum human chorionic gonadotropin (HCG) concentrations. Heterotopic pregnancy was defined as the coexistence of an intrauterine sac and ectopic pregnancy (only one patient was detected).

### Collected data

We collected the data from the fertility center's records. The following variables were considered for the study:

### Baseline characteristics

Maternal and paternal age, female body mass index (BMI), history of EP, previous miscarriage, previous pelvic surgery history, history of tubal factor infertility (TBI), endometriosis, male factor, ovulation factor, basal follicle-stimulating hormone (FSH) and antral follicle count (AFC).

### Treatment characteristics

Gonadotropin-releasing hormone (GnRH) agonist or antagonist protocol (we included only fresh cycles in this analysis), number of mature oocytes retrieved, number of embryos transferred, fresh or frozen embryo transfer, endometrial thickness prior to embryo transfer, sperm concentration and the day of embryo transfer. Cleavage-stage embryo was defined as an embryo transferred in its third day of development (D3), before achieving the blastocyst stage on day five (D5). The ORP and PGT cycles were also analyzed.

### Controlled ovarian stimulation, *in vitro* fertilization, endometrial preparation and embryo transfer

In the GnRH Agonist protocol, triptorelin 0.05 mg daily was started during the mid-luteal phase of the previous cycle. For the GnRH antagonist protocol, we added GnRH antagonist cetrorelix 0.25 mg once the leading follicle reached a diameter of 14 mm. For both protocols, we started treatment with gonadotropin (75-300 IU/daily) on the third day of the menstrual cycle, and was individualized and adjusted according to ovarian response until the leading follicle reached a mean diameter of 18 mm or two or more follicles reached a diameter of 17 mm. The choice of gonadotropin was flexible and considered clinician indication. Recombinant hCG 250 mcg was administered subcutaneously 34-36 hours before ultrasonography-guided oocyte retrieval. In the antagonist protocol, if the patient was at high risk of developing ovarian hyperstimulation syndrome, triggering was performed with GnRH agonist triptorelin 0.2 mg and fresh embryo transfer was not performed (freeze all strategy). Semen was collected through masturbation and prepared by density gradient centrifugation. Some patients underwent Percutaneous Epididymal Sperm Aspiration (PESA) or Testicular Sperm Aspiration (TESA) due to azoospermia.

The Center Intra Cytoplasmic Sperm Injection (ICSI) protocols and laboratory procedures have been described elsewhere (Petracco et al., 2006). Embryos were cultured for 2-6 days, depending on their morphological score on Day 2. The women performed a natural IVF cycle or received estradiol valerate stimulation for endometrium preparation for frozen embryo transfer cycles. During ET, the patient was placed in a lithotomy position and the cervix was exposed using a speculum. Transfer catheter sets were used for all transvaginal ETs, with a standard transfer volume of 5µL. Starting the day after oocyte retrieval and throughout the luteal phase, all patients received vaginal micronized progesterone (600-800 mg daily) (Petracco et al., 2006). The blood sample of beta hCG (β-hCG) subunit was measured two weeks after ET, and transvaginal ultrasound was performed in 3-5 weeks to confirm the clinical diagnosis of pregnancy.

The transfer technique remained unchanged during this 20-year period. Also, when frozen embryo transfers were performed, the freezing technique followed a vitrification protocol. Morphological grade assigned to the embryos was based on criteria from the Latin American Assisted Reproduction Network and was adapted to the clinical protocol. Therefore, the embryo classification was based on blastomere symmetry parameters and the percentage of cytoplasmic fragmentation (REDLARA, 1998). Embryos were graded from I to IV, according to quality. The "good quality" embryos were considered those of grades I and II. The blastocysts were graded according to their size, density, inner cell mass and trophectoderm development, according to the Gardner classification system (Gardner & Schoolcraft, 1999). A grade of "3BB" or higher was defined as "good quality".

### Statistical analysis

Statistical analysis was performed using the Statistical Package for the Social Sciences (SPSS) for Windows, version 22.0 (SPSS Inc., Chicago, IL, USA). The data were expressed as mean ± standard deviation, median with interquartile range, or absolute and relative frequency. Continuous variables were compared using the Student T-test and U-Mann-Whitney test, whereas categorical variables were compared using the Chi-square test or the Fisher's exact test depending on the sample size. A multivariate logistic regression analysis was performed to assess risk factors of EP following IVF-ET. The regression model was adjusted by potential confounders (maternal age, number of mature oocytes, and year of treatment). The null hypothesis was rejected when *p*<.05.

### Ethical Approval

The study has been submitted and was approved by the Research Ethics Committee of the Pontifical Catholic University of Rio Grande do Sul (Protocol 2.769.083). Data was collected from clinical records. All authors signed the Data Use Agreement, preserving patient confidentiality.

## RESULTS

A total of 110 pregnancies (from 106 patients) were ectopic and 1 was heterotopic in a group of 4.666 embryo transfer cycles that resulted in a clinical pregnancy (2.35%). Table 1 depicts the baseline characteristics comparing EP and IUP.

**Table 1.** Baseline characteristics comparison between ectopic and intrauterine pregnancy groups.

Variables	Ectopic	Intrauterine	*p*
Maternal Age (years)	35.0±3.7	34.7±4.1	.525*
Paternal Age (years)	38.6±5.6	37.7±6.1	.222*
Female BMI (kg/m^2^)	22.8±3.4	22.8±3.2	.981*
Number of mature oocytes	10 (2-32)	8 (1-34)	.149^†^
Basal FSH (IU/L)	5.7 (1-11)	6.5 (1-35)	.060^†^
AFC	12 (4-32)	10 (2-47)	.057^†^
Infertility diagnosis %(n/all)** -Tubal factor -Endometriosis -Male factor	35.6 (31/87) 29.9 (26/87) 47.1 (41/87)	21.1 (85/403) 21.1 (85/403) 44.9 (181/403)	.005^‡^ .090^‡^ .723^‡^
Previous History %(n/all)** -Previous ectopic pregnancy -Previous pelvic surgery history -Previous miscarriage	4.6 (4/87) 10.3 (9/87) 13.3 (12/78)	4.5 (18/403) 9.2 (37/403) 6.8 (28/381)	>0.99^‡^ .689^‡^ .050^‡^

BMI: Body mass index, FSH: follicle-stimulating hormone, AFC: antral follicle count.

Values presented as are mean ^±^ standard deviation, n (%), or median (interquartile range).

* Student t test,

† U-Mann–Whitney test,

‡ Fisher exact test.

** patients with missing data were not included in this analysis.

The treatment characteristics, including analysis of endometrial thickness, embryo classification, protocol with GnRH agonist or antagonist or fresh versus frozen ET are presented in Table 2.

**Table 2.** Treatment characteristic comparison between ectopic pregnancy and intrauterine pregnancy groups.

Variables	Ectopic	Intrauterine	*p*
Endometrial thickness, n (%)	n=90	n=397	.935^†^
-<8 -8-12 ->12	4.4 72.2 23.3	5.3 70.8 23.9	
Embryo Classification	n=90	n=409	.905^†^
-Good quality -Poor quality -Mixed	86.7 4.4 8.9	85.8 3.9 10.3	
Day of transfer*	n=65	n=326	.028^‡^
-D3 -D5	69.2 30.8	54 46	
Protocol**	n=61	n=277	.667^‡^
-Agonist -Antagonist	62.3 37.7	58.8 41.2	
Fresh or frozen ET***	n=82	n=397	>0.999^‡^
-Fresh -Frozen	24.4 75.6	25.2 74.8	>0.999^‡^

ET: embryo transfer, good quality:  embryos classified as grades I or II; poor quality: grades III or IV, mixed: when a grade I or II was transferred with a grade III or IV. D3: embryos transferred on third day of development, D5: embryos transferred on day 5. Values are n (%).

† Chi-square test,

‡ Fisher exact test. Patients with missing data were excluded from the analysis - study population delimited in n=.

* Day 3 or Day 5 - day 2 and day 4 transfers were excluded from this analysis,

** only fresh transfer cycles included,

*** excluded cycles using fresh embryos from frozen oocytes.

The results regarding risk factors for EP are presented as a multivariable logistic regression model, adjusted by maternal age, year of treatment and the number of mature oocytes are presented in Table 3.

**Table 3.** Variables for Predicting the risk of ectopic pregnancy after in vitro fertilization-embryo transfer.

Variables	OR (95% CI)	*p*	ORAdj (95% CI)	*p*
Agonist GnRH Protocol	.98 (.89-1.00)	.737	0.72 (.85-1.11)	.727
Day of embryo transfer - D3	1.91 (1.08-3.39)	.025	2.35 (1.27-4.35)	.006
Fresh embryo transfer	1.04 (.60-1.81)	.879	0.86 (.50-1.48)	.596
Endometrial thickness (mm) <8mm 8–12 >12	1 1.21 (.40-3.65) 1.16 (.36-3.73)	- .785 .905	1 1.15 (.38-3.48) 1.06 (.32-3.47)	- .805 .916
Two or more embryos transferred	2.53 (1.12-5.70)	.025	2.26 (1.01-5.21)	.050
Embryo classification Good Quality Poor Quality Mixed	1 1.12 (.36-3.45) .85 (.38-1.89)	- .837 .704	1 1.25 (.40-3.91) 0.89 (.40-1.91)	- .692 .778
Age > 37	1.32 (.80-2.18)	.269	1.43 (.68-3.01)	.339
Endometriosis	1.59 (.9 -2.67)	.077	1.60 (.95-2.70)	.730
Tubal factor	2.07 (1.25-3.41)	.004	2.08 (1.25-3.44)	.04
Previous Miscarriage	2.09 (1.02-4.29)	.044	1.82 (.87-3.81)	.11

OR: odds ratio, ^ADJ^: adjusted, CI: confidence interval, D3: embryos transferred on third day of development, ET: Embryo transfer. Univariate OR and multivariate OR conditional logistic regressions were used to analyze the relationship between the risk factors and ectopic pregnancy. All variables were adjusted in OR adj by female age, year of treatment and number of mature oocytes.

The analysis including PGT cycles showed no statistical difference between the groups. Also, there were no differences in the rates of: use of donor sperm, being an oocyte donor, sperm concentration and the source of spermatozoa obtained (masturbation, PESA or TESA). The different brands of media used in embryo culture or different catheters used in ET did not show any difference between the groups. Data regarding this result are presented in Table 4.

**Table 4.** Additional analysis on Preimplantation genetic test cycles, use of donor sperm, being oocyte donor, sperm characteristics and origin, embryo media and catheter brand.

Characteristic	Ectopic	Intrauterine	*p*
PGT	5.7	2.1	.090*
Oocyte Donor	2.2	4.6	.395*
Use of Donor Sperm	1.1	3.2	.482*
Sperm origin, Sperm concentration Masturbation, Severe Oligospermia <5 million per mL Masturbation, Oligospermia 5-14.9 million per mL Masturbation, Normal sperm Concentration >14.9 million per mL PESA, azoospermia TESA, azoospermia	12.2 17.8 64.4 2.2 3.3	11.5 10 70.9 3.7 3.9	.300^†^
Media Brand CSC™ (HSA), (Irvine Scientific, U.S.) G1 media (Vitrolife) G2.2 media (Vitrolife) Global (LifeGlobal, U.S.) HTF (Modified Human Tubal Fluid Medium) 15% (Irvine Scientific) HTF (Modified Human Tubal Fluid Medium) 10% (Irvine Scientific) SSM™ (Single Step Medium) (Irvine Scientific, U.S.)	11.4 1.1 3.4 39.8 39.8 1.1 3.4	24.9 3.4 1.7 34.2 32 .5 3.2	.102^†^
Catheter Brand FRYDMAN SOFT Rocket Thinwall Embryo Transfer Catheter Cook Sydney Guardia Embryo Transfer Catheters	27.8 1.1 64.4 6.7	21.8 .2 67.8 10.1	.311^†^

PGT: Preimplantation Genetic Tests; PESA: Percutaneous Epididymal Sperm Aspiration; TESA: Testicular Sperm Aspiration due to azoospermia. Values are %.

* Fisher exact test,

† Chi-square test.

Finally, we analyzed the proportion of ORP in the EP and IUP groups (10% [10/100] versus 3.1% [13/422], *p*=.005). The risk of developing EP in the ORP was 3.49 (95% CI 1.48-8.22, *p*=.004) and after OR adjustment it was 2.85 (95% CI 1.08-7.51, *p*=.034).

## DISCUSSION

The overall EP rate in the present study was consistent with that reported previously for ART pregnancies (Clayton et al., 2006). Our results showed that tubal factor infertility, previous miscarriage, D3 embryo transfers and transfer of two or more embryos seems to be associated with higher risk for EP after IVE-ET.

The occurrence of an EP is a dramatic event in the reproductive history of a woman as it represents the end of a desired pregnancy that often requires surgical treatment and can result in the definitive loss of a fallopian tube or a serious permanent impairment of its function (Ticconi et al., 2018). The incidence of EP in IVF patients is usually reported as 2%-5%, with some papers showing an incidence as high as 11% (Liu et al., 2019). The mechanism of this relatively high incidence is still not clear. Also, regarding ART techniques, studies have postulated some theories to decrease EP risk.

In the present study, regarding baseline characteristics, there were no differences in maternal and paternal ages, maternal BMI, number of mature oocytes, basal FSH, and antral follicle count. Also, there were no differences in male factor, ovulation factor, previous EP, and previous pelvic surgery history. On the other hand, important considerations can be made regarding specific aspects, as discussed below.

### Endometriosis

Studies have associated endometriosis with increased EP risk (Perkins et al., 2015). Similarly, Hjordt Hansen et al. (2014) compared women with and without endometriosis finding that EP was almost twice more frequent in the endometriosis group. Factors influencing the higher incidence include the reflux of endometrial tissue fragments into the fallopian tube, providing uterine cavity epithelial characteristics and the inflammatory aspects of endometriosis. Even though endometriosis was not statistically different in our study, the rates were higher in the EP group which could be associated with limited statistical power.

### Tubal factor infertility

Tubal factor infertility is a known factor for EP after ART treatment (Jee et al., 2009; Malak et al., 2011). When studying patient's past medical histories, we found that patients with TBI had a two-fold higher risk of developing an EP.

### History of miscarriage

In the present study, a previous history of miscarriage (at least one) appeared to be associated with a two-fold risk of EP. However when we adjusted for confounders factors the OR lost significance. A previous retrospective study investigated the association between recurrent miscarriage (RM) and EP. They found that the EP rate was 2.31 higher in RM compared to control women; even though their study has excluded ART patients, those findings are similar to our findings.

Both EP and RM share several characteristics such as similar ranges of incidence, tendency to be a repetitive problem in the same patient, occurring more frequently at similar gestational age, and in women of older age. Although it is evident that EP and RM are different conditions, the possibility of an association between RM and EP cannot be completely ruled out (Ticconi et al., 2018).

### Endometrial thickness

We did not find an association between endometrial thickness and risk of developing EP. It is important to state that in our population only six patients presented an endometrial thickness of 6 mm or less. The proportion of patients with endometrial thickness > 12 was similar between the groups. Liu et al. (2019) found that an endometrial thickness >12 mm was a strong protective factor against EP (OR 0.27; 95% CI 0.13-0.56, *p*<.001). The possible explanation is that with the decline of endometrial thickness, the endometrial receptivity decreases, leading to a higher likelihood of embryo implantation outside the uterine cavity (Ma et al., 2017).

### GnRH antagonist and GnRH agonist protocol

In a comparative study of fresh versus frozen embryo transfers, the authors found that in fresh embryo transfer cycles the odds of EP were 52% higher in GnRH antagonist cycles, as compared with the luteal GnRH agonist protocol. The authors hypothesized that extrapituitary GnRH might play a more important role in early implantation processes (Londra et al., 2016). In our study, we did not find that difference, and the rates of agonist or antagonist were similar between the groups.

### D3 and D5 embryo transfer

In the present study, we found a 2.3 fold higher risk of EP after D3 transfer. This is consistent with previous findings (Zhang et al., 2017). Blastocysts have been demonstrated to have a higher overall implantation potential, a larger diameter, and a shorter time prior to implantation, and these factors are related to a lower risk of retrograde travel into the Fallopian tubes, decreasing an ectopic implantation (Smith et al., 2013). Also, the primary direction of the uterine contractile waves following ovulation are from the cervix towards the fundus, and these retrograde uterine contractions decrease progressively in frequency and amplitude by day 6 or 7 following hCG trigger. Therefore, embryo transfer on D5 could lower the likelihood of ectopic implantation compared with D3 embryo transfer. Other studies failed to find this association, but it could be due to limited statistical power (Milki & Jun, 2003; Smith et al., 2013).

### Frozen and fresh embryo transfer

Londra et al. (2015) found that the odds of EP were 65% lower in women who had a frozen compared with a fresh transfer in autologous cycles. Their findings support the hypothesis that ovarian hyperstimulation in IVF may generate a uterine environment that increases the risk of endometrial implantation failure and an abnormally located implantation compared with embryo transfers without ovarian hyperstimulation. In our study we did not find any difference between fresh versus frozen ET.

### Multiple-embryo transfer

Multiple studies suggested higher EP risk with multiple-embryo transfer (Clayton et al., 2006; Perkins et al., 2015). We found that the transfer of two or more embryos was associated with a 2.2 fold higher risk of EP. Since this is a 20-year study, we must highlight that it was common to perform D3 transfer with three and sometimes even more embryos. This practice is less common today and acknowledging the high implantation potential at the blastocyst stage, current recommendations favor the transfer of fewer embryos at the blastocyst stage versus at cleavage stage (Londra et al., 2015).

### Ovarian reserve

Previous studies also associated lower ovarian reserve with higher EP risk (Lin et al., 2017; Kim et al., 2019). It could be related to lower oocyte quality and sub-optimal hormonal environment in ovaries with decreased capacity. In our study, the patients presented similar ages, FSH, AFC count and mature oocytes rates and there were no differences between the groups.

### Oocyte recipient patients

The incidence of EP in ORP was 3.0 times higher. Most studies that analyzed EP risk excluded ORP (Rombauts et al., 2015; Muller et al., 2016; Lin et al., 2017; Liu et al., 2019). However, a study found opposite results: the proportion of EP was higher among IVF cycles with autologous oocytes than among cycles with oocytes from a donor (Londra et al., 2015). The number of patients that we included in this analysis was limited, 10 patients were ORP in the EP group and 13 in the IUP group; however, the difference between the groups was significant, in univariate and multivariate analyses, which seems to be an interesting finding. Finally, in this group of patients choosing blastocyst, single embryo transfer may be a reasonable clinical practice.

### Ectopic Pregnancy Algorithm

**Figure 2:**
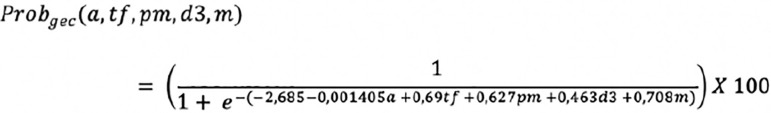
Algorithm to predict the probability of Ectopic Pregnancy After IVF, where a=Patient´s Age, tf=Tubal Factor, pm=Previous Miscarriage, d3=Third Day Transfer, m=More Than One Embryo Transferred.

Considering the variables that might be related to EP, we developed an algorithm using the patient's age, tubal infertility history, miscarriage history, multiple embryo transfer and D3 transfer. Using data from this study, and applying them to this algorithm, we predicted the probability of EP in this group of patients with an area under the curve of 64 (Figure 2).

### Study limitation

The main limitation of this study was its retrospective nature. In 20 years, different strategies (such as ovarian stimulation and laboratory protocols, laboratory environment, method of embryo culture, form of embryo and gametes' manipulation) have been used, and new technologies were incorporated. To reduce this impact, we adjusted findings for the year when the treatment was performed. We also calculated if different catheters used in embryo transfer or different media used in embryo culture resulted in higher EP risk, as different brands were used throughout these years, and we found no difference between the groups. We did not analyze the gonadotropin protocol used, since this is a private practice clinic and clinicians are allowed to use gonadotropin according to their preference. However previous studies found no impact on the type, number of days or dosage of gonadotropin used on EP risk (Fang et al., 2015; Weiss *et al*., 2016; Liu *et al*., 2019). Other variables that may have affected the association with EP include the use of ultrasound on embryo transfer, and smoking history. Information regarding surgeon's ease or difficulty performing the ET (including the use of a tenaculum) was not available.

## CONCLUSION

The results of this study illustrated that tubal factor was an independent risk factor for EP following IVF. Previous miscarriage was associated with higher EP risk, but the difference was not statistically significant after adjusting for confounders. However, in the researcher's opinion, the difference was clinically significant. Transfer in cleavage state and more than one embryo transferred were associated with greater EP risk. Thus, we suggest that in high-risk patients, such as those with tubal infertility history and miscarriage history, single blastocyst transfer may be a reasonable approach to diminish EP risk. Oocyte recipient patients should be carefully evaluated, as this study found greater EP risk in those patients, even though the sample was limited.

## References

[r1] Bu Z, Xiong Y, Wang K, Sun Y (2016). Risk factors for ectopic pregnancy in assisted reproductive technology: a 6-year, single-center study. Fertil Steril.

[r2] Cheng LY, Lin PY, Huang FJ, Kung FT, Chiang HJ, Lin YJ, Lan KC (2015). Ectopic pregnancy following in vitro fertilization with embryo transfer: A single-center experience during 15 years. Taiwan J Obstet Gynecol.

[r3] Clayton HB, Schieve LA, Peterson HB, Jamieson DJ, Reynolds MA, Wright VC (2006). Ectopic pregnancy risk with assisted reproductive technology procedures. Obstet Gynecol.

[r4] Fang C, Huang R, Wei LN, Jia L (2015). Frozen-thawed day 5 blastocyst transfer is associated with a lower risk of ectopic pregnancy than day 3 transfer and fresh transfer. Fertil Steril.

[r5] Gardner DK, Schoolcraft WB (1999). Culture and transfer of human blastocysts. Curr Opin Obstet Gynecol.

[r6] Hjordt Hansen MV, Dalsgaard T, Hartwell D, Skovlund CW, Lidegaard O (2014). Reproductive prognosis in endometriosis. A national cohort study. Acta Obstet Gynecol Scand.

[r7] Jee BC, Suh CS, Kim SH (2009). Ectopic pregnancy rates after frozen versus fresh embryo transfer: a meta-analysis. Gynecol Obstet Invest.

[r8] Kim SW, Kim YJ, Shin JH, Kim H, Ku SY, Suh CS, Kim SH, Choi YM (2019). Correlation between Ovarian Reserve and Incidence of Ectopic Pregnancy after In Vitro Fertilization and Embryo Transfer. Yonsei Med J.

[r9] Lin S, Yang R, Chi H, Lian Y, Wang J, Huang S, Lu C, Liu P, Qiao J (2017). Increased incidence of ectopic pregnancy after in vitro fertilization in women with decreased ovarian reserve. Oncotarget.

[r10] Liu X, Qu P, Bai H, Shi W, Shi J (2019). Endometrial thickness as a predictor of ectopic pregnancy in 1125 in vitro fertilization-embryo transfer cycles: a matched case-control study. Arch Gynecol Obstet.

[r11] Londra L, Moreau C, Strobino D, Garcia J, Zacur H, Zhao Y (2015). Ectopic pregnancy after in vitro fertilization: differences between fresh and frozen-thawed cycles. Fertil Steril.

[r12] Londra L, Moreau C, Strobino D, Bhasin A, Zhao Y (2016). Is the type of gonadotropin-releasing hormone suppression protocol for ovarian hyperstimulation associated with ectopic pregnancy in fresh autologous cycles for in vitro fertilization?. Fertil Steril.

[r13] Ma NZ, Chen L, Dai W, Bu ZQ, Hu LL, Sun YP (2017). Influence of endometrial thickness on treatment outcomes following in vitro fertilization/intracytoplasmic sperm injection. Reprod Biol Endocrinol.

[r14] Malak M, Tawfeeq T, Holzer H, Tulandi T (2011). Risk factors for ectopic pregnancy after in vitro fertilization treatment. J Obstet Gynaecol Can.

[r15] Milki AA, Jun SH (2003). Ectopic pregnancy rates with day 3 versus day 5 embryo transfer: a retrospective analysis. BMC Pregnancy Childbirth.

[r16] Muller V, Makhmadalieva M, Kogan I, Fedorova I, Lesik E, Komarova E, Dzhemlikhanova L, Niauri D, Gzgzyan A, Ailamazyan E (2016). Ectopic pregnancy following in vitro fertilization: meta-analysis and single-center experience during 6 years. Gynecol Endocrinol.

[r17] Perkins KM, Boulet SL, Kissin DM, Jamieson DJ, National ART Surveillance (NASS) Group (2015). Risk of ectopic pregnancy associated with assisted reproductive technology in the United States, 2001-2011. Obstet Gynecol.

[r18] Petracco A, Azambuja R, Okada L, Michelon J, Oliani A, Badalotti M (2006). Comparison of embryo quality between sibling embryos originating from frozen or fresh oocytes. Reprod Biomed Online.

[r19] REDLARA (1998). Red Latino Americana de Reproduccion Assistida, ed. Manual de Procedimientos Laboratorio de Reproduccion Asistida.

[r20] Refaat B, Dalton E, Ledger WL (2015). Ectopic pregnancy secondary to in vitro fertilisation-embryo transfer: pathogenic mechanisms and management strategies. Reprod Biol Endocrinol.

[r21] Rombauts L, McMaster R, Motteram C, Fernando S (2015). Risk of ectopic pregnancy is linked to endometrial thickness in a retrospective cohort study of 8120 assisted reproduction technology cycles. Hum Reprod.

[r22] Smith LP, Oskowitz SP, Dodge LE, Hacker MR (2013). Risk of ectopic pregnancy following day-5 embryo transfer compared with day-3 transfer. Reprod Biomed Online.

[r23] Ticconi C, Capogna MV, Martelli F, Borelli B, Bruno V, Ergasti R, Sorge R, Piccione E, Pietropolli A (2018). Ectopic pregnancy in women with recurrent miscarriage. J Obstet Gynaecol Res.

[r24] Weiss A, Beck-Fruchter R, Golan J, Lavee M, Geslevich Y, Shalev E (2016). Ectopic pregnancy risk factors for ART patients undergoing the GnRH antagonist protocol: a retrospective study. Reprod Biol Endocrinol.

[r25] Zhang B, Cui L, Tang R, Ding L, Yan L, Chen ZJ (2017). Reduced Ectopic Pregnancy Rate on Day 5 Embryo Transfer Compared with Day 3: A Meta-Analysis. PLoS One.

